# Quality of Life and Life Satisfaction in Former Athletes: A Systematic Review and Meta-Analysis

**DOI:** 10.1007/s40279-019-01163-0

**Published:** 2019-08-19

**Authors:** Stephanie Filbay, Tej Pandya, Bryn Thomas, Carly McKay, Jo Adams, Nigel Arden

**Affiliations:** 1 Centre for Sport, Exercise and Osteoarthritis Research Versus Arthritis, Nottingham, UK; 2 Nuffield Department of Orthopaedics, Rheumatology and Musculoskeletal Sciences, University of Oxford, Oxford, UK; 3 Faculty of Biology, Medicine and Health, University of Manchester, Msnchester, UK; 4 Rotorua Hospital, Lakes District Health Board, Rotorua, New Zealand; 5 Department for Health, Centre for Motivation and Health Behaviour Change, University of Bath, Bath, UK; 6 School of Health Sciences, Faculty of the Environment and Life Sciences, University of Southampton, Southampton, UK

## Abstract

**Background:**

Sport participation has many physical and psychosocial benefits, but there is also an inherent risk of injury, subsequent osteoarthritis and psychological challenges that can negatively impact quality of life (QOL). Considering the multifaceted impacts of sport participation on QOL across the lifespan, there is a need to consolidate and present the evidence on QOL in former sport participants.

**Objective:**

To evaluate QOL and life satisfaction in former sport participants, and determine what factors are associated with QOL and life satisfaction in this population.

**Methods:**

Eight electronic databases were systematically searched in July 2018 to retrieve all articles that evaluated QOL or life satisfaction in former sport participants. Two authors independently screened titles/abstracts and full texts, extracted data, and appraised methodological quality using a modified Downs and Black Checklist. Random-effects meta-analysis estimated pooled mean and 95% confidence intervals (Cis) for Mental Component Scores (MCS) and Physical Component Scores (PCS) derived from the SF-12, SF-36, VR-12 and VR-36 measures. MCS and PCS were pooled for all former sport participants, as well as professional- and collegiate-athlete subgroups. Data that were inappropriate for meta-analysis (i.e. EQ-5D, PROMIS and life-satisfaction outcomes) were collated and reported descriptively.

**Results:**

Seventeen articles evaluated QOL or life satisfaction in a total of 6692 former athletes [eight studies (*n* = 4255) former professional athletes; six studies (*n* = 1946) former collegiate athletes; two studies (*n* = 491) included both] with a mean age ranging from 21 to 66 years. Most studies were cross-sectional (15 of 17 articles) and 12 studies had a moderate risk of bias (*n* = 1 high-risk, *n* = 4 low-risk). Unpublished data were provided for five studies. Meta-analysis of seven studies resulted in a pooled PCS mean (95% CI) of 50.0 (46.6–53.3) [former professional athletes from two studies: 46.7 (42.1–51.2), former collegiate athletes from five studies: 51.2 (48.4–53.9)] and a pooled MCS of 51.4 (50.5–52.2) [former professional athletes: 52.7 (51.3–54.2), former collegiate athletes: 50.9 (50.0–51.8)]. Factors associated with worse QOL or life satisfaction in former athletes included involuntary retirement from sport (three studies), collision/high-contact sport compared with low/no-contact sport (three studies), three or more concussions compared with no/fewer concussions (two studies), increased body mass index (BMI) (worse PCS, three studies), and osteoarthritis or musculoskeletal issues (worse PCS and MCS, three studies; worse PCS but not MCS, two studies).

**Conclusions:**

Former athletes had similar PCS and better MCS, compared to general-population norms. Former athletes with impaired PCS reported better MCS than population norms, highlighting the need to use an instrument that differentiates between physical and mental components of QOL in former sport participants. Factors associated with worse QOL that may explain between-study variation include involuntary retirement, collision/high contact sports, concussion, BMI and osteoarthritis.

**PROSPERO:**

CRD42018104319.

**Electronic supplementary material:**

The online version of this article (10.1007/s40279-019-01163-0) contains supplementary material, which is available to authorized users.

## Key Points

**Table:** 

In former collegiate and professional athletes, physical components of QOL were similar and mental components of QOL were better than general population norms, on average.
Reported QOL varied greatly between studies, which may be explained by factors associated with worse QOL including involuntary retirement, collision/high contact sport, concussion, BMI and osteoarthritis.
There was a discordance between physical and mental components of QOL in former athletes, highlighting the importance of using measurement instruments that differentiate between physical and mental components of QOL in this population.
Evaluating life-satisfaction in addition to QOL in former athletes would be beneficial, as this allows former athletes to assess the quality of their lives on the basis of their own unique set of criteria.

## Background

In a given month, every second resident in the UK aged over 13 years participates in sport [1]. Sport participation is associated with a range of physical health benefits, including a reduction in all-cause mortality, superior lifespan longevity and a reduced risk of diabetes, cardiovascular disease and osteoporosis [2–4]. However, after retiring from sport, former athletes can adopt an inactive lifestyle [5–7], which places them at the same or higher risk for developing chronic disease as the inactive general population [8, 9] and is related to reduced life satisfaction [10]. Sport participation also brings an inherent risk of injury [11, 12], which can cause a cascade of negative emotions for an athlete [13], impaired quality of life (QOL) in athletes compared to their uninjured peers [14–17] and reduced physical activity levels irrespective of functional recovery [18].

Sports injury can also have long-lasting physical and psychological impacts that persist across the lifespan, with potential impacts on health-related QOL (HRQoL) and life satisfaction. Sport-related concussion is related to reduced HRQoL and depression in former athletes [19–23], and joint injury places athletes at risk of developing osteoarthritis [24, 25]. Former elite athletes have a higher prevalence of osteoarthritis compared to the general population and people in other occupations [26]. Osteoarthritis in former sport participants has been associated with distress, sleep disturbance, adverse alcohol use, symptoms of common mental disorders and reduced QOL [27, 28]. The high prevalence of osteoarthritis may explain the greater levels of pain and physical impairment in former elite athletes compared with an aged-matched general population [29]. Although living with joint pain and osteoarthritis is common in former athletes, previous qualitative research suggests that this does not necessarily correspond with poor reported QOL or life satisfaction, and that the positive psychosocial impacts of sport may partly counteract the negative physical implications of sport participation [30].

Sport participation for people of all ages is associated with an array of psychosocial health benefits including resilience, improved mental health, a sense of belonging, higher levels of self-efficacy, reduced stress, enhanced coping and positivity [31–37]. Considering these benefits, it is not surprising that people participating in sport report better HRQoL than the general population [17, 38]. The psychosocial and HRQoL benefits associated with sport participation are greater than those associated with physical activity participation alone [31, 39]. However, athletes may struggle psychologically and socially when transitioning out of sport. Reported challenges include a change in athletic identity, a loss of camaraderie and minimal support systems [40, 41]. Adapting to life post-sport appears more difficult when an athlete is retiring involuntarily due to injury or deselection [41]. Forced retirement from sport can result in high levels of negative emotions, social exclusion, a loss of identity and a sense of betrayal [8]. In contrast, voluntary retirement and achievement of sporting goals are associated with a positive experience of transition from sport [8, 42]. Research suggests that accomplishment and positive sporting memories could benefit QOL and life satisfaction in later life, irrespective of joint pain or osteoarthritis [30, 43].

Most research has focused on athletes’ QOL during their career or during their retirement transition period [8, 33, 40, 42, 44]. QOL and life satisfaction in former sport participants is less understood, and considering the potential for sport participation to have both positive and negative impacts on QOL and life satisfaction across the lifespan, there is a need to consolidate the evidence on QOL and life satisfaction in former sport participants. Factors with potential to impact these constructs after retirement from sport include reason for retirement, type of sport, injury and concussion history, activity levels, osteoarthritis and chronic pain. However, it is not currently clear what factors are related to a better or worse QOL and life satisfaction after retirement from sport. Such information may inform strategies to enhance the positive impacts of sport across the lifespan.

The aims of this systematic review were to (1) evaluate QOL and life satisfaction in former sport participants and (2) determine what factors are associated with QOL and life satisfaction in former sport participants.

## Methods

This systematic review used the Preferred Reporting Items for Systematic Reviews and Meta-Analyses (PRISMA) guidelines for conducting and reporting systematic reviews [45], and the protocol for this review was prospectively registered on PROSPERO (CRD42018104319, 24 July 2018).

### Search Strategy

We systematically searched eight electronic databases in July 2018 to retrieve all relevant articles: Scopus, MEDLINE, CINAHL, EMBASE, The Cochrane Library, PubMed, PsycINFO and SPORTDiscus. The search strategy retrieved articles that included a term from each of the following three concepts in the title and/or abstract, or as an associated MeSH (Medical Subject Headings) term:

- Concept 1. Sport (included terms related to ‘sport’ or ‘athlete’ as well as a comprehensive list of sports);
- Concept 2. Former (included the terms ‘former*’, ‘past*’, ‘retire*’ or ‘ex-*’); and
- Concept 3. Quality of life (including terms relevant to QOL, wellbeing and life satisfaction as well as commonly used instruments evaluating these constructs).

The complete search strategy is presented in Electronic Supplementary File 1. All titles and abstracts were independently screened for eligibility by two authors (TP, BT). All articles with the possibility of being eligible progressed to full-text screening to confirm inclusion or exclusion. Following this, the list of articles for inclusion was cross-checked between authors, and any discrepancies were resolved by a third author (SF). Finally, the reference lists of eligible articles were screened to ensure all relevant articles had been retrieved through the database search.

### Eligibility Criteria

To be included in the review, articles had to meet the following predefined eligibility criteria:

#### Inclusion Criteria

- Quality of life or life satisfaction was assessed using a standardised valid measure in former sport participants (e.g. former professional athletes, former college athletes or former recreational sport participants);
- Article published in English

#### Exclusion Criteria

- Studies limited eligibility of participants based on a pre-specified QOL or life-satisfaction score (e.g. only participants with impaired QOL were recruited);
- Qualitative studies, case-reports, review articles or conference abstracts;
- Only a non-validated question(s) that may pertain to QOL, well-being or life satisfaction was used;
- Only part (i.e. not all domains or items) of a validated measure of QOL or life-satisfaction was used;
- Intervention studies that did not report QOL or life satisfaction data pre-intervention (or provide this data on request).

Studies of former elite or college athletes were included in this review irrespective of current recreational sport participation. The rationale for this was that very few studies in former athlete samples report current sport or physical activity participation. Despite this, such studies may contribute valuable information aligning with the aims of this review.

The Cochrane Collaboration recommends that reviewers contact the original investigators of eligible studies to request additional information relevant to the review that cannot be extracted from the manuscript [46]. If a study evaluated QOL or life-satisfaction in eligible participants but did not report descriptive data in the manuscript, or only a subgroup of participants was eligible but QOL or life-satisfaction was not reported specifically for this subgroup, the authors were contacted requesting this data. The article was excluded if the authors did not respond following two attempts at contact or did not provide the information necessary for inclusion.

Where two eligible articles reported data from the same sample or a subset of the same sample, both papers were included in the review if contributing novel information that aligned with the aims. Where two studies from the same cohort reported QOL or life-satisfaction data in a format enabling meta-analysis (i.e. mean (standard deviation (SD))) or mean (95% confidence interval (CI))), only the paper with the greatest sample size contributed to meta-analysis. If only one paper reported data in an appropriate format, this paper contributed to the meta-analysis, irrespective of sample size.

### Terminology

#### Sport

Sport was operationally defined as “a subset of exercise that can be undertaken individually or as a part of a team, where participants adhere to a common set of rules or expectations, and a defined goal exists” [3].

#### Health-Related QOL

HRQoL is a multi-dimensional construct, encompassing aspects of overall QOL that affect either physical or mental health [47]. HRQoL has been described as an individual’s subjective assessment of the physical, psychological and social domains of health [48].

#### Life Satisfaction

Life satisfaction has been defined as “a judgmental process, in which individuals assess the quality of their lives on the basis of their own unique set of criteria” [49].

### Data Extraction

Two authors (TP, BT) independently extracted study information, participant characteristics and descriptive data from each paper. The data were cross-checked between authors and any inconsistencies resolved through discussion with a third author (SF). Study characteristics included aim, year, country, sample size, study design, eligibility criteria and comparison group(s). Participant characteristics included sex, body mass index (BMI), age at follow-up(s), age at sport cessation, type of sport, standard of sport, length of sport participation and time since ceasing sport participation.

### Methodological Appraisal

The methodological quality and risk of bias of each article were evaluated independently by two authors (TP, BT) and any discrepancies were resolved by a third author (SF). Methodological quality was evaluated using a modified version of the Downs and Black Checklist for the Assessment of Methodological Quality of Randomised and Non-Randomised Studies [50]. This tool includes domains evaluating the quality of reporting, external validity, bias and confounding. It is appropriate for methodological appraisal of a variety of study designs; however, the full criteria are often modified to align with specific review aims, especially when RCTs are not appropriate to address a study question. Items that were not applicable as per the aims of this review were excluded and the wording of one item was modified to align with the aims of this review (item 11), resulting in a total of 14 items. The full modified Downs and Black Checklist and interpretation of each item is presented in Electronic Supplementary File 2. Each of the 14 items were equally weighted, and assigned a score of ‘1’ for meeting a criterion, or ‘0’ if the criterion was not met or this could not be determined from the information provided. The methodological appraisal score ranged from 0 (lowest possible methodological quality) to 14 (highest possible methodological quality). To aid in interpretation, we classified study quality and risk of bias based on the proportion of criteria met: < 50% = low quality, high risk of bias; 50–75% = fair quality, moderate risk of bias; 76–100% = high quality, low risk of bias.

### HRQoL Outcome Measures

*The Optum SF™ Health Surveys* and generic health surveys capture reliable and valid information about functional health and well-being across a wide variety of age, treatment and disease groups [51]. All *Optum SF™ Health Surveys* (e.g. SF-36v1, SF-36v2, and SF-12) measure the same eight health domains, enabling the calculation of two summary scores: the Physical Component Score (PCS) (physical functioning, bodily pain, general health perceptions and physical role limitation), and the Mental Component Score (MCS) (vitality, emotional role functioning, social role functioning and mental health). Norm-based scoring is recommended for these measures over the alternative 0–100 scores, which are associated with floor and ceiling effects and do not allow for comparison between domains [51].

The 1998 USA population values used for norm-based scoring were derived from administration of the SF36v1 and SF36v2 to a population sample (*n* = 6742), with random instrument allocation [52, 53]. The norm-based scoring algorithm employs a linear *T* score transformation with a mean of 50 and a standard deviation of 10, derived from 1998 USA general population norms. For all scales and summary measures, group mean scores below 47 can be interpreted as being below the average range for the general population [51].

There are several surveys derived from the *Optum SF™ Health Surveys.* The Veterans RAND 36 Item Health Survey (VR-36) is an adaptation of the SF-36, where yes/no responses for two domains (‘physical role limitation’ and ‘emotional role functioning’) were replaced with 5-point response choices. The Veterans RAND 12-Item Health Survey (VR-12) was adapted from VR-36 and is similar to the SF-12 but replaces yes/no responses from two items on the SF-12 with 5-point response choices. The VR-36 and VR-12 also contain two additional items that assess how the patient’s mental and physical health has changed over time [54]. The same norm-based scoring algorithm employing a linear *T* score transformation with a mean of 50 and a standard deviation of 10 derived from 1998 USA general population norms can be employed to calculate domain scores and PCS and MCS summary scores for VR-36 and VR-12 measures, enabling comparison with the *Optum SF™ Health Surveys.*

*The EuroQol*-*5D (EQ*-*5D)* comprises five items evaluating mobility, self-care, usual activities, pain/discomfort and anxiety/depression. Each item is scored on a 3- or 5-point scale (depending on the 3-level or 5-level version), and summed to provide an overall ‘health-status’ score. Weighted scores are used to calculate a utility index, where 0 represents ‘death’ and 100 represents ‘perfect health’ [55].

*The Patient Reported Outcome Measure Information System (PROMIS*) includes over 300 measures of physical, mental and social health for use in the general population and with individuals with chronic conditions. PROMIS includes a combination of individual items, fixed sets of short-forms addressing specific domains; and profiles combining fixed selection of short-forms from multiple domains. Global Health is a profile comprising ten items, allowing physical and mental health summary scores to be calculated [56]. Scores range from 0 to 100 and are calibrated using a *T*-score metric with a USA general population mean of 50 and a standard deviation of 10 [56]. For the physical and mental health summary scores, a higher score indicates better HRQoL. Higher scores for ‘sleep’, ‘anxiety’, ‘depression’, ‘fatigue’ and ‘pain interference’ indicate poorer health, while higher scores for ‘physical function’ and ‘satisfaction with participation in social roles’ indicate better health.

### Statistical Analysis

Using a random-effects meta-analysis, a pooled summary mean and 95% CI for MCS and PCS scores (i.e. from SF-12, SF-36v1, SF-36v2, VR-12, VR-36 health measures) was calculated using the R statistical software program [57]. Studies were weighted according to variance within and between studies. A pooled summary mean and 95% CI for MCS and PCS scores were calculated for all studies (i.e. all former sport participants) and for distinct sub-groups of former sport participants (former professional athletes and former collegiate athletes). Combining other QOL or life-satisfaction data using meta-analysis was inappropriate due to the small number of studies using comparable outcomes. Instead, these were reported descriptively. Factors that were investigated in relation to QOL in former sport participants were collated and reported descriptively when investigated in more than one study.

If QOL data were reported separately in two subgroups of an eligible sample [58–62], subgroups were combined using a formula from The Cochrane Handbook for Systematic Reviews of Interventions to obtain mean and SD estimates for the combined groups [46].

## Results

**Fig. 1:**
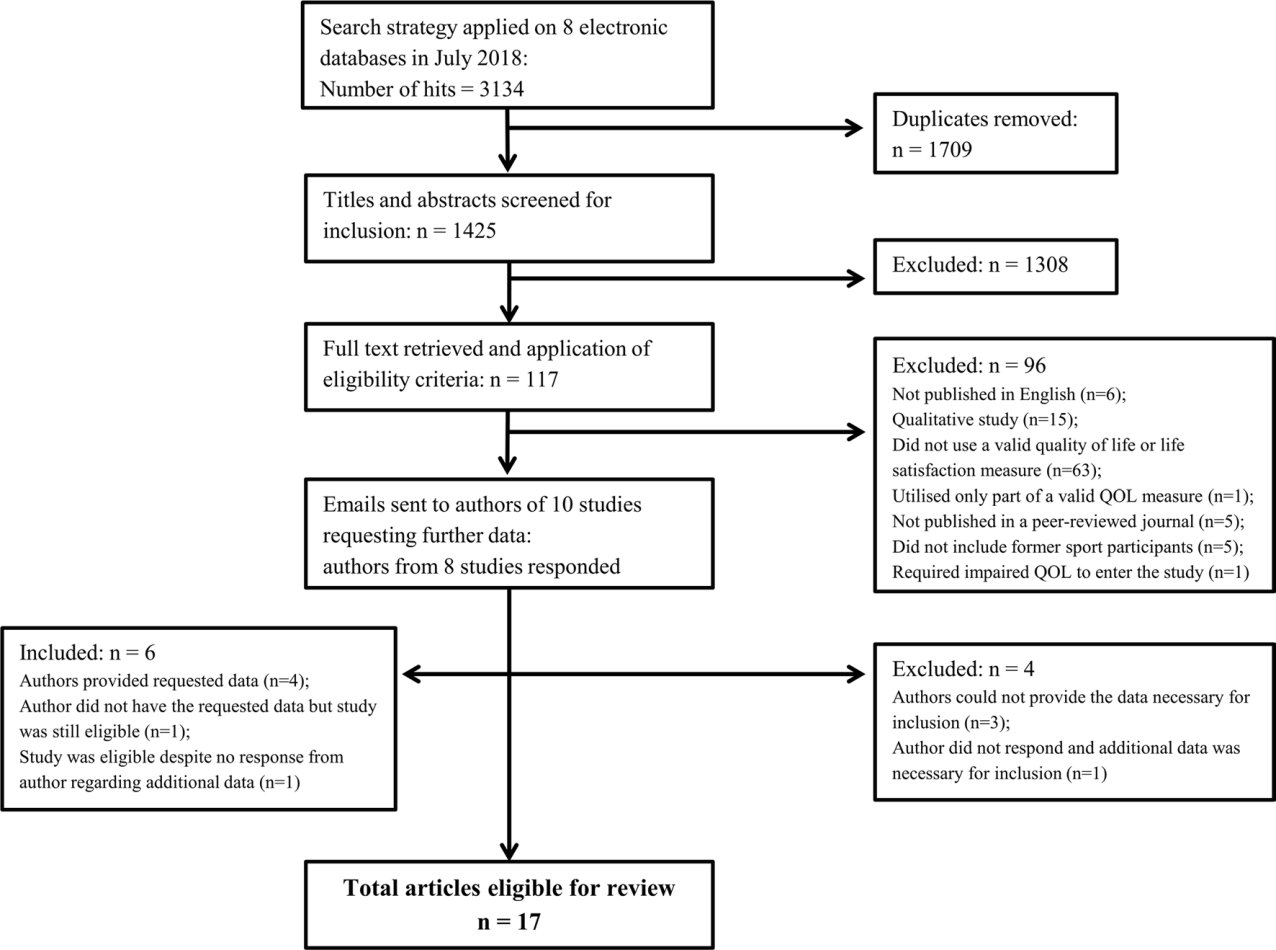
Search strategy. *QOL* quality of life

The systematic search performed on eight databases yielded a total of 3134 articles (Fig. 1). After removal of 1709 duplicate articles, a further 1308 articles were excluded through screening of titles and abstracts. This resulted in 117 articles for full-text retrieval and further eligibility screening. At this stage, a further 96 articles were excluded (see Fig. 1 for reasons for exclusion). Additional data were requested via email from authors of ten studies. Authors from eight studies responded; additional data were provided for five studies [25, 63–66] and four articles were excluded since data necessary for inclusion were not provided (Fig. 1). This resulted in 17 eligible articles for review. Two articles analysed HRQoL in a subset of the same sample of participants [67, 68]. The author of three studies confirmed via email that these articles addressed independent samples [63–65].

### Methodological Appraisal

There was a 93.3% agreement between items following initial assessment by two independent reviewers; all differences were resolved during a consensus meeting. Quality appraisal scores ranged from 3 (lowest quality) to 12 (highest quality) (mean score 9 SD 2). One study was classified as having a high risk of bias [43], four studies were classified as low risk of bias [21, 59–61] and all other studies were classified as moderate risk (Table 1). Three items were met by no more than one study, highlighting three common biases within study designs. Only one study described the characteristics of patients lost to follow-up (or non-responders), no studies provided sufficient information to determine that subjects who were prepared to participate were representative of the entire population from which they were recruited, and only one study reported sufficient power to detect a clinically important effect where the probability value for a difference being due to chance was < 5%. All other items were met by over 50% of studies (Table 2).

**Table 1:** Methodological appraisal scores

References	Methodological appraisal item														% Met	Risk of bias^a^
	1	2	3	4	5	6	7	8	9	10	11	12	13	14		
Arliani [67]	1	1	1	1	1	1	0	1	1	U	1	1	0	1	64	Mod
Arliani [68]	0	1	1	1	1	1	0	0	1	U	1	1	0	0	57	Mod
Backmand [69]	1	1	1	1	1	1	0	0	1	U	0	1	1	U	64	Mod
Barbosa Filho [70]	1	1	1	1	1	1	0	1	1	U	0	1	1	U	71	Mod
Davies [25]	1	1	1	1	1	1	0	0	1	U	1	U	1	0	64	Mod
Gouttebarge [66]	1	1	1	1	1	0	0	1	1	U	1	1	0	U	64	Mod
Guskiewicz 2007 [23]	1	1	1	1	1	1	0	0	1	U	1	1	1	U	71	Mod
Kerr [21]	1	1	1	1	1	1	0	1	1	U	1	1	1	U	79	Low
Kerr [60]	1	1	1	1	1	1	0	1	1	U	1	1	1	0	79	Low
Kleiber [43]	0	1	0	0	0	0	0	1	0	0	0	1	0	0	21	High
Martin [71]	1	1	1	0	1	1	0	0	1	U	1	1	0	0	57	Mod
Nicholas [59]	1	1	1	1	1	1	0	1	1	U	1	1	1	0	79	Low
Simon [63]	1	1	1	1	1	1	0	1	1	U	0	1	0	U	64	Mod
Simon [64]	1	1	1	1	0	1	0	0	1	U	0	1	1	0	57	Mod
Simon [65]	1	1	1	1	1	1	0	0	U	U	0	1	0	0	50	Mod
Sorenson [61]	1	1	1	1	1	1	1	1	1	0	1	1	1	0	86	Low
Turner [72]	1	1	0	0	1	1	0	1	U	U	1	1	1	0	57	Mod
% Met	88	100	88	82	88	88	6	59	82	0	65	88	59	6		

^a^To aid in interpretation, we classified study quality and risk of bias based on the proportion of criteria met: < 50% = low quality, high risk of bias; 50–75% = fair quality, moderate risk of bias; 76–100% = high quality, low risk of bias

1 = Yes (criteria met); 0 = No (criteria not met); *U* Unable to determine (assigned a score of 0)

**Q1**. Is the hypothesis/aim/objective of the study clearly described?; **Q2** Are the main outcomes to be measured clearly described in the introduction or methods section? **Q3.** Are the characteristics of the participants included in the study clearly described? **Q4.** Are the distributions of principal confounders in each group of subjects to be compared clearly described? **Q5**. Are the main findings of the study clearly described? **Q6**. Does the study provide estimates of the random variability in the data for the main outcomes? **Q7.** Have the characteristics of patients lost to follow-up (or non-responders) been described? **Q8.** Have actual probability values been reported (e.g. 0.035 rather than < 0.05) for the main outcomes except where the probability value is less than 0.001? **Q9**. Were the subjects asked to participate in the study representative of the entire population from which they were recruited? **Q10.** Were those subjects who were prepared to participate representative of the entire population from which they were recruited? **Q11.** Was the sample appropriately described with regards to sport-related characteristics? **Q12**. Were the main outcome measures used accurate (valid and reliable)? **Q13**. Was there adequate adjustment for confounding in the analyses from which the main findings were drawn? **Q14.** Did the study have sufficient power to detect a clinically important effect where the probability value for a difference being due to chance < 5%

**Table 2:** Study characteristics

Article	Design	Origin	*N* = former athletes^a^	Sex (% male)	Age (years)	BMI (kg/m^2^)	Type of sport participation	Years since sport retirement	Length of sport participation (years)
Arliani et al. [67]	CS	Brazil	27	100	45.7 ± 5.9 (range 20–55)	25.73 ± 3.15	Professional soccer	NR	≥ 5 years, mean 14.9
Arliani et al. [68]	CS	Brazil	16	100	Median 44.38 SD 4.97	Median 25.96 SD 3.58	Professional soccer	NR	≥ 5 years, mean 15.5
Backmand et al. [69]	LC	Finland	758	100	Mean 66.3 (range 46–92)	NR	Elite Finish sport (Olympics, European/World Championships or intercountry competitions 1920–1965)	NR	NR
Barbhosa Filho et al. [70]	CS	Brazil	186	64	50.5 ± 6.5 (range 40–64)	25.77 ± 3.41	Southern Brazil multisport medallists 1960–2006	5–10 (26%); 11–15 (13%); > 15 60%)	NR
Davies et al. [25]	CS	UK	259	100	60.1 ± 16.1	28.1 ± 3.7	Oxford University, Cambridge University or English international Rugby	NR	22.2 ± 5.3 (range 1–43)
Goutterbarge et al. [66]	CS	English-, French-, Spanish-speaking countries	396	10	36 ± 6	NR	Professional Soccer (first or second highest national league)	5 ± 4	11 ± 5
Guskiewickz et al. [23]	CS	USA	2552	100	53.8 ± 13.4	NR	Professional American football	24.7 ± 13.7	6.6 ± 3.6
Kerr et al. [21]	CS	USA	797	47	< 24 (5%); 25–34(39%); 35–44(41%); 45 + (15%)	< 25 (56%); 25–29.9 (33%); > 30 (11%)	29 different NCAA Division I Collegiate sports (11.5% played professionally)	14.5	Minimum 1 season
Kerr et al. [60]	CS	USA	204	100	< 34 (15%); 34–37 (77%) 38 + (8%)	< 25 (9%); 25–29.9 (44%); 30–34.9 (34%) 35–39.9 (8%) 40 + (5%)	Collegiate American football (did not play professionally)	15	Minimum 1 season
Kleiber et al. [43]	CS	USA	427	100	NR	NR	Collegiate basketball and football	NR	NR
Martin et al. [71]	LC	Australia	16	NR	21.6 ± 5.1 (range 14–36)	NR	Elite athletes from 23 different sports	2.1 (range 1–3)	NR
Nicholas et al. [59]	CS	USA	36	100	62 ± 3 (range 58–75)	NR	American Football, 1969 Super Bowl winning team	32 ± 3 (range 24–36)	8.3 ± 3.8
Simon et al. [63]	CS	USA	232	72	53.4 ± 7.1 (range 40–65)	NR	Division 1 Collegiate sport; 22% played professionally	NR	College sport: 11% = 2;17% = 3; 60% = 4; 12% = 5 Professional sport: 22% = 1–7; 78% = 0
Simon et al. [64]	CS	USA	374	67	52.4 ± 7.5	NR	Division 1 Collegiate collision (American football, 94%) contact (7 sports), or limited-contact (8 sports) sports	NR	College sport: ≤10% = 2; ≤ 10% = 3; 75% = 4; ≤ 10% = 5 Professional sport: 30% = 1–10; 70% = 0
Simon et al. [65]	CS	USA	100	60	53.1 ± 7.4 (range 40–65)	NR	24 Division 1 collegiate sports (30% American football)	NR	NR
Sorenson et al. [61]	CS	USA	44	73	45.6 ± 16.2	26.3 ± 4.2	13 Division 1 collegiate sports	NR	3.0 ± 1.3
Turner et al. [72]	CS	UK	284	100	56.1 ± 11.8	NR	Professional soccer	NR (retirement age 32.3 ± 4.7 years)	13.5 ± 5.3

Results are reported as mean ± standard deviation, unless specified otherwise. Percentages for subgroups are given (i.e. for age, BMI, years since retirement, length of sport participation) where no alternative data were reported and insufficient data was reported for calculation of combined estimates

^a^*n* represents the number of former sport participants within each study, in some cases this is a sub-group of the full study sample

*CS* cross-sectional, *LC* longitudinal cohort, *UK* United Kingdom, *USA* United States of America, *NR* not reported, *NCAA* National Collegiate Athletic Association

### Study and Participant Characteristics

HRQoL or life satisfaction was evaluated in a total of *n* = 6692 former athletes, including *n* = 4255 former professional athletes (eight studies); *n* = 1946 former USA division 1 collegiate athletes (six studies); and *n* = 491 athletes who played at either a USA collegiate or UK university level and/or professionally (two studies). The most common study design was cross-sectional (15 of 17 articles) and performed in the USA (nine of 16 studies). Nine studies (56%) included only male participants, six studies (38%) included between 47% and 73% male participants and one study did not describe participant sex (Table 2). The mean age of former athletes ranged from 21 to 66 years with 12 studies (75%) reporting a mean or median age between 45 and 66 years. Only five studies reported mean or median BMI data (mean (SD) range 25.7–28.1 (3.2–4.2) kg/m^2^). Six studies reported time since retiring from sport (range mean 2–32 years) and seven studies reported length of sport participation (range mean 3–22 years) (Table 2).

#### Outcome Measures

HRQoL was evaluated with the SF-36 in seven articles (version 1 [68, 73] and version 2 [23, 59, 64, 65, 70]) and three studies used either the SF-12 [61], VR-12 [21] or VR-36 [60]. Two articles from the one study reported only SF-36 domain scores [68, 73], whereas all other articles reported MCS and PCS scores. Two studies utilised the EQ-5D (EQ-5D-3L [62] and EQ-5D-5L [74]) and two studies utilised variations of the PROMIS [63, 75]. Life satisfaction was evaluated in three studies, using the Satisfaction With Life Scale (SWLS) [71], Life Satisfaction Index-version A (LSI-A) [43] and Allardt’s Life Satisfaction Scale [69].

### Quality of Life in Former Sport Participants

#### HRQoL Evaluated with SF-36, VR-36, VR-12 and SF-12 Measures

PCS and MCS scores from seven studies were available for pooling in meta-analysis. One study did not use norm-based scoring (SF-36 scores, PCS: 81.0 SD 18.9; MCS: 80.2 SD 16.0) and was excluded from meta-analysis [70]. Two articles from a sub-set of the same sample of former professional soccer players only reported SF-36 domain scores, precluding inclusion in meta-analysis (median domain scores: PF: 90, RP: 50, BP: 62, GH: 87, V: 75, SF: 87.5, RE: 100, MH: 84 [67]; mean(SD) domain scores PF: 88.8(14.9), RP: 53.1(39.7), BP: 65.8(26.9), GH: 88.3(12.9), V: 73.8(17.3), SF: 83.6(24.9), RE: 68.8(41.2), MH: 82.8(16.4) [68]).

#### Physical Component Scores

**Fig. 2:**
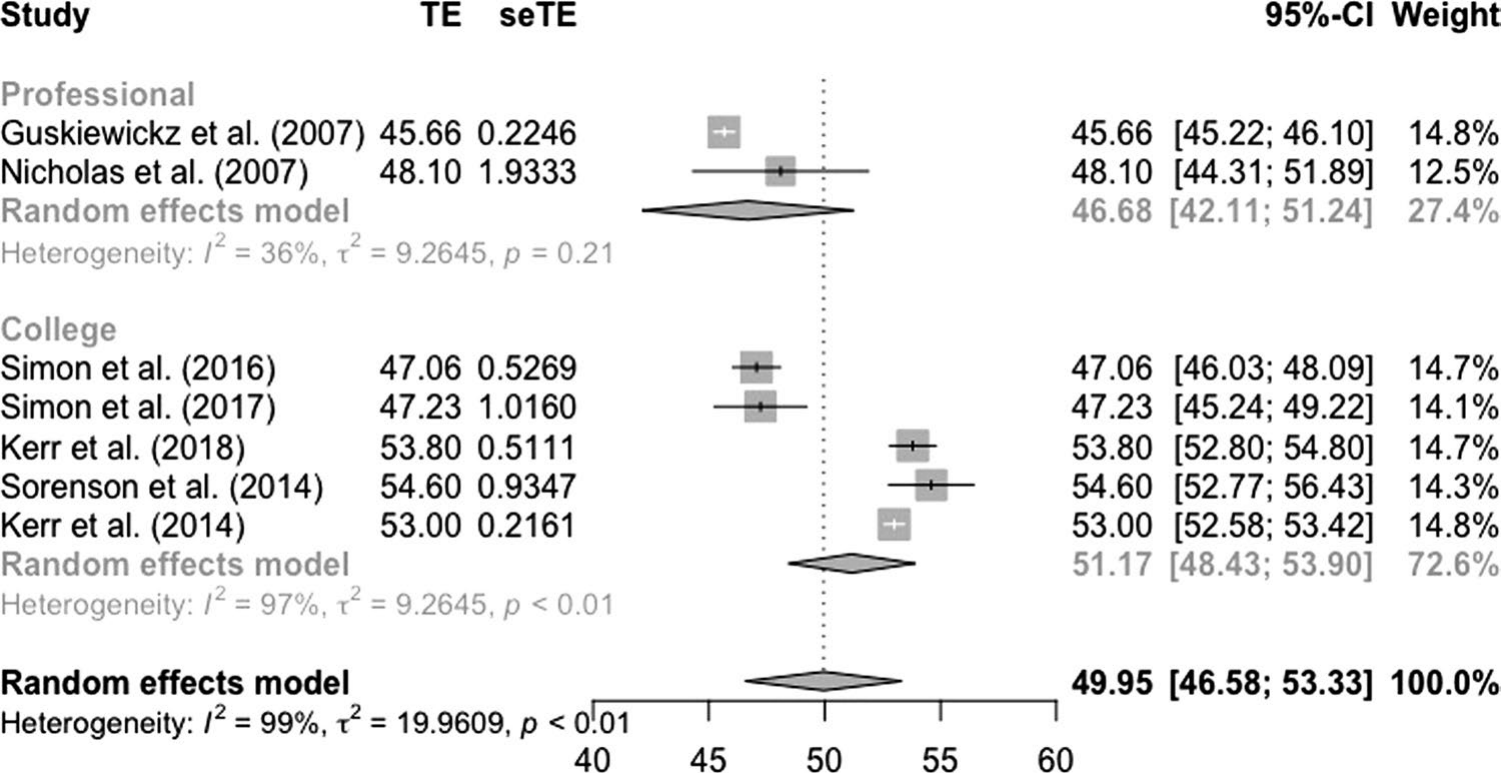
Random-effects meta-analysis of HRQoL physical component scores in former athletes

Pooling PCS scores using random-effects meta-analysis resulted in a pooled mean (95% CI) of 50.0 (46.6–53.3) for all studies of former sport participants (Fig. 2). Subgroup analyses resulted in a pooled mean of 46.7 (42.1–51.2) for studies in retired professional athletes (all retired American football players), and a pooled mean of 51.2 (48.4–53.9) for former Division 1 USA Collegiate athletes (Fig. 2).

#### Mental Component Scores

**Fig. 3:**
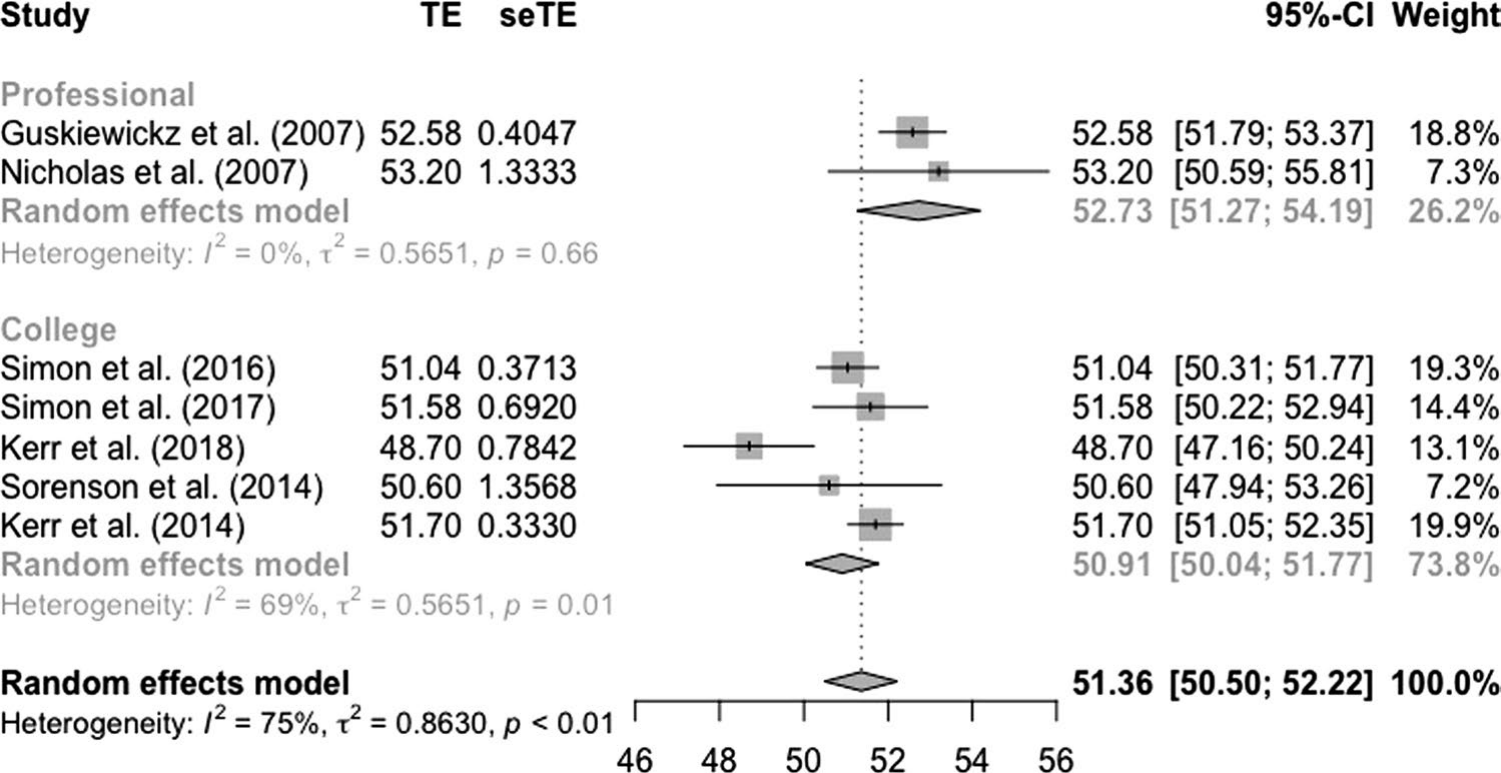
Random-effects meta-analysis of HRQoL mental component scores in former athletes

Random-effects meta-analysis resulted in a pooled mean MCS score of 51.4 (50.5–52.2) for all studies, a pooled mean of 52.7 (51.3–54.2) for studies in retired professional American football players, and a pooled mean of 50.9 (50.0–51.8) for former Division 1 USA Collegiate athletes (Fig. 3).

#### EQ-5D

Davies et al. provided unpublished EQ-5D-5L data from former rugby union players (*n* = 247) and reported a mean (SD) EQ-5D utility score of 0.783 (0.147) [25]. Turner et al. reported a mean (SD) EQ-5D-3L utility score of 0.70 (0.28) in former professional UK soccer players [72].

#### PROMIS

Simon et al. provided mean (SD) data on request for seven domain scores from the PROMIS in 232 former Division I collegiate athletes: Physical function: 37.5 SD 13.2; Anxiety: 44.9 SD 7.2; Depression: 51.7 SD 10.4; Fatigue: 51.5 SD 8.9; Sleep: 85.5 SD 3.1; Satisfaction with social role: 55.3 SD 14.1; Pain interference: 55.2 SD 11.1 [63]. Goutterbarge et al. provided unpublished PROMIS data on request for 396 former professional soccer players (global physical health: mean 51.0 SD 7.6 and global mental health: 51.5 SD 8.2) [66].

#### Life Satisfaction

Three studies used different measures to evaluate life satisfaction in former athletes. Backmand et al. used Allardt’s Life Satisfaction Scale in 758 former elite Finish athletes who reported mean (standard error) scores of: 7.79 (0.22) (Endurance sports); 8.20 (0.17) (Power combat sports); 8.42 (0.22) (Power/individual sports); 8.23 (0.16) (Team sports); 7.88 (0.30) (Shooting) [69]. Kleiber et al. used the Life Satisfaction Index-A (LSI-A) in 427 former college basketball/football players, and reported mean values of 2.90/2.97 (no recognition received/some recognition received); 2.80/2.94 (injury ended career/injury did not end career); 2.90/2.95 (started < half of games/started > half of games) [43]. Martin et al. used the Satisfaction With Life Scale (SWLS) in 16 former elite athletes, who reported a mean (SD) score of 27.19 (5.47) [71].

### Factors that may be Associated with a Better or Worse Quality of Life in Former Sport Participants

#### Age and Sex

Of the three studies that investigated the relationship between age and HRQoL, one study with low risk of bias found no relationship [70] and two studies (with moderate and low risk of bias) found an association between age and HRQoL in former athletes [21, 61]. Kerr et al. found that compared to a sex- and aged-matched USA population sample, former college athletes reported better MCS scores if they were male aged 35–44 years (53.3 SD 7.8 vs. 50.4 SD 9.6) or female aged 18–24 (49.6 SD 11.2 vs. 44.4 SD 11.4), 25–34 (51.5 SD 9.1 vs. 47.1 SD 10.7) or 35–44 (51.9 SD 9.2 vs. 47.8 SD 10.4) years. Women aged 35–54 years also reported better PCS scores than a sex- and aged-matched USA population sample (35–44 years (52.9 SD 6.5 vs. 51.6 SD 8.6) and 45–54 years (52.5 SD 6.7 vs. 48.5 SD 10.5)) [21]. Sorenson et al. found former college athletes who were aged ≥ 43 years reported worse PCS scores than those aged ≤ 42 years (52.5 SD 6.6 vs. 56.9 SD 4.8), and *better* MCS scores than their younger counterparts (51.5 SD 9.6 vs. 49.6 SD 8.4) [61]. Two studies (with low and moderate risk of bias) found no association between HRQoL and sex [21, 70].

#### Body Mass Index

Of the three studies investigating the relationship between BMI and PCS scores, three studies (with low [21, 60] or moderate risk of bias [70]) reported an association between increasing BMI and worse PCS scores (BMI < 25.0 kg/m^2^: PCS mean 54.0 SD 5.3 vs. BMI ≥ 30 kg/m^2^: PCS mean 50.5 SD 6.8, *p* < 0.001 [21]; for every five-point increase in BMI the prevalence of having a PCS < 50 increased by 75% (95% CI 38–122) [60]; unadjusted regression standardised coefficient score − 0.32, *p* < 0.001 [70]).

Only two studies reported results regarding the relationship between BMI and MCS scores. One moderate risk of bias study reported an association between variables (unadjusted regression standardised coefficient score − 0.18, *p* = 0.01 [70]) and one low risk of bias study found similar MCS scores between groups (< 25.0 kg/m^2^: 51.6 SD 9.4 vs. 25.0–29.9 kg/m^2^: 51.9 SD 9.5 vs. ≥ 30 kg/m^2^: 51.2 SD 9.5 [21]).

#### Age That Participants Started Playing Sport

One study (with low risk of bias) in former collegiate athletes found no association between HRQoL and the time at which participants started playing sport [21]. In contrast, a study in former collegiate American football players (with low risk of bias) found that after controlling for concussion history and BMI, for every additional 3 years earlier that someone started playing football, PCS scores decreased by 1.0 (95% CI 0.0–2.0) [60]. This study found that age at the time of commencing football was not associated with MCS scores [60].

#### Reason for Retirement from Sport

Three studies found worse HRQoL (moderate risk of bias study) [21] or life-satisfaction (high and moderate risk of bias studies) [43, 71] in former athletes who retired involuntarily. Individuals who ended collegiate sport participation due to injury reported worse PCS scores (50.3 SD 8.1 vs 53.5 SD 5.5) [21], similar MCS scores (52.8 SD 9.1 vs. 51.4 SD 9.5) [21], and lower life satisfaction [43] compared to those who did not cease collegiate sport due to injury. Martin et al. also found athletes who retired voluntarily reported greater life satisfaction (median SWLS score: 33 vs. 25) than those who retired involuntarily (e.g. due to injury or deselection) [71].

#### Type of Sport

Three studies (1 low [21] and 2 moderate [64, 69] risk of bias) found worse HRQoL or life-satisfaction scores in former collision/high contact athletes compared with former low/no contact athletes. Former collegiate collision or high-contact athletes reported worse PCS scores (collision, 51.4 SD 7.3; high contact, 51.9 SD 6.5; low/no contact, 53.6 SD 5.6) and similar MCS scores (53.0 SD 8.7; 51.4 SD 11.0; 51.5 SD 9.0) compared to former collegiate low/non-contact athletes [21]. Simon et al. found that former collegiate collision-athletes reported worse PCS (mean difference 12.7) and MCS scores (mean difference 8.6 points) than former limited-contact athletes. In this study, former collision athletes also reported worse PCS and MCS scores compared with contact athletes, but a smaller difference was observed compared to limited-contact athletes [64]. Similarly, Backmand et al. found that power sport (i.e. boxing, wrestling, weightlifting, throwers) and team-sport (i.e. soccer, ice hockey, basketball) athletes reported worse life-satisfaction than former shooters and endurance sport athletes [69].

#### Standard of Sport Participation

One low risk of bias study reported similar MCS and PCS scores between former collegiate athletes who had played professionally, and those who had not [21]. A moderate risk of bias study found that Former Division I collegiate athletes reported worse scores on several HRQoL domains compared to former college students who did not participate in Division I sport but regularly played intramurals, club, or other recreational activity during college (PROMIS mean difference: physical function: 17.51; depression: 7.31; fatigue: 5.25; sleep disturbances: 5.88; pain interference: 10.17) [63]. Domain scores for ‘anxiety’ and ‘satisfaction with social roles’ were similar between groups. Former Division 1 athletes in this study had more major injuries, chronic injuries, daily limitations, physical activity limitations and a higher prevalence of osteoarthritis than the comparison group [63].

#### Concussion History

Two studies investigated the relationship between concussion history and HRQoL in former collegiate athletes. One low risk of bias study found that former athletes with a history of three or more concussions reported worse PCS scores (50.9 SD 7.6 vs. 53.4 SD 5.5) and similar MCS scores (50.4 SD 10.5 vs. 52.3 SD 8.9) compared to former athletes with no history of concussion [21]. In contrast, a low risk of bias study found former collegiate football players with three or more concussions reported worse MCS (mean difference = 7.7 (3.4–11.9)) and similar PCS (mean difference = 2.2 (0.6–5.1)) scores, compared to those with no history of concussion; and worse MCS (mean difference = 6.1 (2.7 to 9.4)) and PCS (mean difference = 2.7 (0.6 to 4.8)) scores compared to those with a history of one or two concussions [60].

#### Osteoarthritis and Musculoskeletal Health

Of the five studies that investigated the relationship between osteoarthritis or musculoskeletal issues and HRQoL, three studies (with moderate risk of bias) found worse HRQoL (physical and mental components) in former athletes with osteoarthritis/musculoskeletal issues compared to former athletes without these conditions. This included PROMIS scores (Global Physical Health 47.6 vs. 52.7; Global Mental Health 49.9 vs. 52.2 [66]), EQ-5D utility scores (0.58 SD 0.31 vs. 0.81 SD 0.19 [62]) and associations with worse MCS and PCS scores in a multivariable model (sports injury that affects current daily living (yes = 1); unadjusted regression standardised coefficient score PCS: − 0.53, *p* < 0.00; MCS: − 0.22, *p* = 0.003 [70]).

In contrast, two studies found impaired PCS but not MCS scores in former athletes with pain or osteoarthritis [59, 65]. A small study found PCS scores were impaired in former NFL Super Bowl athletes with arthritis (did not specify type of arthritis) (PCS with arthritis: 42.6 SD 11.7 vs. without arthritis: 55.9 SD 4.6) and MCS scores were similar between those with and without arthritis (53.1 SD 8.9 vs. 53.3 SD 6.4) [59]. A moderate risk of bias study found worse PCS scores in former athletes with a history of knee surgery and knee osteoarthritis (41.1 SD 6.8), compared to former athletes with no history of knee surgery and no osteoarthritis (51.1 SD 6.4) and former athletes with a history of knee surgery without knee osteoarthritis (47.4 SD 7.5) [65]. MCS scores were similar between groups (49.3 SD 9.5, 52.6 SD 8.5 and 53.3 SD 10.1, respectively).

#### Factors Investigated in One Study

Factors investigated in one study that were found to have no association with HRQoL or life satisfaction included ethnicity [21], education level [70], use of non-prescription medicine [70], time since retiring from sport [70], significant injury history [59], starting ≥ 50% of college basketball or football games [43], recognition of achievement [43], health guidance from coaches [70] and time spent walking or performing moderate physical activity in the past week [70].

Factors investigated in one study that were associated with HRQoL included use of prescription medicine (worse PCS and MCS scores [70]), no employment (worse PCS but not worse MCS scores [70]), a lower income (worse MCS but not PCS scores [70]) and a greater amount of vigorous physical activity during the past week (better PCS and MCS scores [70]).

## Discussion

Compared to population norms [51], former athletes reported similar physical aspects of HRQoL (PCS scores), on average. However, there was distinct variation in PCS values between studies (Fig. 2). Compared with the general population, three studies reported worse PCS scores and three studies reported better PCS scores. Our findings indicate that there are a number of factors that could explain contrasting PCS scores between different samples of former athletes, including differences in the proportion of participants who played collision or contact sports, BMI, different reasons for retiring from sport, concussion history and osteoarthritis. This is also supported by the finding that former athletes with no history of concussion [60] or no osteoarthritis [59] reported better PCS scores than the general population.

In contrast, mental aspects of HRQoL (pooled MCS scores) were better in former professional and collegiate athletes compared to the general USA population [51]. Interestingly, former athletes who reported PCS scores similar [59] or worse [58, 64, 65] than the general population still reported better MCS scores. Additionally, two out of three studies reporting better PCS scores than the general population reported similar or worse MCS scores [60, 61]. This suggests a discordance between physical and mental aspects of HRQoL in former sport participants. Similarly, Davies et al. found physical-related domains of health status were impaired in former rugby players compared to the general UK population, but found no difference in anxiety/depression scores [25]. Additionally, Simon et al. found PROMIS scales of physical function and pain interference were impaired in former athletes compared to the general population; in contrast, anxiety and satisfaction with social roles were higher than population norms [63].

A key contributor to impaired physical aspects of HRQoL in former athletes is osteoarthritis [59, 62, 65, 66, 70]. However, the relationship between osteoarthritis and the mental aspects of HRQoL is less clear, since two studies found osteoarthritis was associated with worse PCS but not MCS scores [59, 65]. Furthermore, collision/contact sport was associated with worse PCS, but not MCS scores, 15 years after collegiate sport participation [21]. In contrast, a large USA population based study (*n* = 1,003,388) found that people with arthritis were more likely to have physically and mentally unhealthy days (including stress, depression and emotional problems) compared to aged-matched individuals without arthritis [76]. This suggests that the discordance between mental and physical components of HRQoL observed in this review, may be specific to former athletes. Backmand et al. found that compared to an aged and residence matched general population sample, former athletes were more satisfied with their lives [69]. Competitive sport participation has been associated with higher levels of mental toughness [77] and increased pain coping and resilience [30]. High levels of resilience are associated with enhanced adaptive coping and adjustment to musculoskeletal pain [30, 78, 79]. Feelings of pride and accomplishment in sport, and social networks developed through years of sport participation, has further potential to positively influence mental components of HRQoL after sport retirement, despite an increased prevalence of musculoskeletal pain and osteoarthritis in former athletes [30].

One of the key findings of this review is that there are differences in physical and mental components of HRQoL in former athletes. Common measures of HRQoL and health status poorly differentiate social and mental health from physical disability [80]. Although the SF-36 is designed to give PCS and MCS summary scores, some items blend physical and mental aspects of HRQoL which can result in measurement error and erroneous conclusions [80, 81]. The EQ-5D is another measure that has been used in former athletes, but largely evaluates physical aspects of health (four of five domains measure physical aspects; pain/discomfort; mobility; limitation in usual activities, self-care). Thus, this instrument may not be appropriate for use in former athlete populations. There is a need to evaluate the measurement properties of HRQoL measures for use in former athletes, where differentiating between physical and mental components of HRQoL should be a key priority. In the meantime, it would be advantageous to select measures of HRQoL for use in former athletes that distinguish between physical and mental components of HRQoL. There may also be benefits in supplementing measures of HRQoL with assessment of life satisfaction, which enables former athletes to evaluate their overall life satisfaction, taking into account both the physical and mental aspects of QOL.

### Strengths, Limitations and Future Research Recommendations

This is the first systematic review to investigate HRQoL and life-satisfaction in former sport participants. One of its key strengths was the retrieval and use of unpublished data from multiple studies, enabling pooling of data for meta-analysis. This study has produced new and important knowledge to improve current understanding of HRQoL in former athletes. Due to limitations in study designs, however, we categorised former professional and former collegiate athletes as ‘former sport participants’. Unfortunately, studies did not evaluate current sport participation and physical activity levels, thus a proportion of participants may still be participating in non-professional or non-collegiate sports. Future studies should ensure current sport participation and physical activity levels are reported. A better understanding of HRQoL in former sport-participants of all playing standards (including recreational sport participants), who are no longer participating in any sport, is needed. Another consideration is that several former collegiate athlete studies included a proportion of individuals who went on to play professionally, whilst other studies did not evaluate or report the proportion who played professionally. This limited our ability to compare HRQoL between former professional and collegiate athletes. There were also insufficient homogenous studies to enable cross-cultural comparisons or comparisons of HRQoL between former athletes from contrasting sports. Additionally, other sport-related details that were poorly or under-reported included BMI, years since retirement from sport and length of sport participation. Another knowledge gap that became apparent during this review was the scarcity of literature investigating HRQoL in former female sport participants; nine articles included only male participants, in comparison to no all-female studies. Furthermore, multiple factors that may be related to HRQoL in former athletes were investigated in only one study (including post-sport employment and income, physical activity levels, injury history, education and time since sport-retirement), highlighting a need for further research. Additionally, longitudinal studies evaluating changes in HRQoL over time in former athletes would provide valuable knowledge to aid the interpretation of study findings. Finally, only three studies had investigated life satisfaction in former sport participants and each study used a different instrument, highlighting the timely need for further studies on life satisfaction in former sport participants.

## Conclusions

On average, former athletes had similar physical aspects of HRQoL and reported better mental aspects of HRQoL, compared to the general population. However, there was discordance between physical and mental aspects of HRQoL; studies reporting the lowest PCS scores reported the highest MCS scores, and vice versa. This relationship is not typical of the general population and may be unique to former athletes, highlighting the importance of evaluating both physical and mental components of HRQoL in former sporting groups. There was distinct variation in HRQoL between studies, which may be explained by playing collision/contact sports, higher BMI, involuntary retirement from sport, a history of concussion and osteoarthritis, which were associated with worse HRQoL in former athletes. These findings demonstrate that sport participation can have long-term physical consequences that negatively impact QOL. However, sport participation may also have positive mental impacts that persist beyond sport-retirement and enhance QOL. Strategies to reduce negative physical impacts and enhance positive mental impacts of sport participation are needed to optimise athlete health and wellbeing across the lifespan.

## Electronic supplementary material

Below is the link to the electronic supplementary material.

Supplementary material 1 (PDF 594 kb)

Supplementary material 2 (PDF 428 kb)
