# Supplementary material for: Quality of Life and Life Satisfaction in Former Athletes: A Systematic Review and Meta-Analysis
Source: Sports Med. 2019 Aug 19;49(11):1723–38. doi: 10.1007/s40279-019-01163-0 (PMC6789047; doi:10.1007/s40279-019-01163-0)
Supplement: Supplementary file 1 — Supplementary material 1 (PDF 594 kb) [file 40279_2019_1163_MOESM1_ESM.pdf]

Electronic Supplementary File 1. Full search strategy, formatted for Scopus

Article title: Quality of life and life satisfaction in former athletes: a systematic review and meta-analysis

Authors: Stephanie Filbay*, Tej Pandya, Bryn Thomas, Carly McKay, Jo Adams, Nigel Arden

***Corresponding author details**: Centre for Sport, Exercise and Osteoarthritis Research Versus Arthritis; Nuffield Department of Orthopaedics, Rheumatology and Musculoskeletal Sciences, University of Oxford, Oxford, United Kingdom. Email: stephanie.filbay@uq.net.au

((TITLE-ABS-KEY("Quality of life" OR qol OR *qol OR qols OR qol* OR "life quality" OR wellbeing OR wellness OR well-being OR "life satisfaction" OR sf-8 OR sf-12 OR sf-36 OR "short-form 36" OR "short-form 12" OR "short-form 8")) or (TITLE-ABS-KEY("short form 36" OR "short form 12" OR "short form 8" OR "Sickness Impact Profile" OR "Nottingham Health Profile" OR "Health Utilities Index" OR RAND OR QWB OR EQ-5D* OR EQ5D* OR "Athlete Life Quality Scale" OR ALQS)) or (TITLE-ABS-KEY("Flourishing Scale" OR "Trojan Lifetime Champions" OR "mental component score*" OR "physical component score*" OR "health status" OR "life quality"))) AND ((TITLE-ABS-KEY(sport* OR athlete* OR athletic* OR archery OR *ball OR Badminton OR Baseball OR Basketball OR Bandy OR Biathlon OR Bobsleigh OR *Boarding OR "Body Building" OR Bowling OR *Boxing OR "Bull Fight*" OR Canoe* OR Cricket OR Curling OR Cycling OR *cycling)) or (TITLE-ABS-KEY(Dance* OR Dancing OR Decathlon OR Diving OR Equestrian* OR Fencing OR Football* OR Gridiron OR Golf OR Gymnast* OR *racing OR Handball OR *Hockey OR Judo OR "Ju Jitsu" OR Karate)) or (TITLE-ABS-KEY(Kayak* OR "Kung-Fu" OR Lacrosse OR "lawn bowls" OR Luge OR Pentathlon OR "Martial Arts" OR "Mountain Biking" OR marathon OR Mountaineering OR Netball OR "Nordic combined" OR "Race walking" OR Polo OR Racquetball OR "Rock Climbing" OR Rodeo)) or (TITLE-ABS-KEY(Rowing OR Rugby OR Sailing OR Shooting OR Ski OR skiing OR skier OR *Skating OR "Snow Board*" OR Shooting OR Soccer OR Softball OR Skeleton OR Squash OR Running OR "ultimate Frisbee" OR Surfing OR Swimming OR *Tennis OR Taekwondo OR "Track and Field")) or (TITLE-ABS-KEY(Trampolining OR Triathlon OR Volleyball OR "Water Polo" OR "Weight Lifting" OR Wrestling))) AND (TITLE-ABS-KEY(former* or past* or retired or retire* or ex-*))
