# Supplementary material for: Quality of Life and Life Satisfaction in Former Athletes: A Systematic Review and Meta-Analysis
Source: Sports Med. 2019 Aug 19;49(11):1723–38. doi: 10.1007/s40279-019-01163-0 (PMC6789047; doi:10.1007/s40279-019-01163-0)
Supplement: Supplementary file 2 — Supplementary material 2 (PDF 428 kb) [file 40279_2019_1163_MOESM2_ESM.pdf]

Electronic Supplementary File 2. Modified Downs and Black Criteria for Quality Appraisal

Article title: Quality of life and life satisfaction in former athletes: a systematic review and meta-analysis

Authors: Stephanie Filbay*, Tej Pandya, Bryn Thomas, Carly McKay, Jo Adams, Nigel Arden

***Corresponding author details**: Centre for Sport, Exercise and Osteoarthritis Research Versus Arthritis; Nuffield Department of Orthopaedics, Rheumatology and Musculoskeletal Sciences, University of Oxford, Oxford, United Kingdom. Email: stephanie.filbay@uq.net.au

Scoring: YES = (1); NO = (0) Unable to determine: UTD = (0)

CRITERIA	EXPLANATION/ INTERPRETATION
1. Is the hypothesis/aim/objective of the study clearly described?	Must be explicit
2. Are the main outcomes to be measured clearly described in the introduction or methods section?	**NO =** If a main outcome is first mentioned in the results section<br>**YES =** if all primary outcomes are described in methods or introduction (i.e. reproducible).
3. Are the characteristics of the participants included in the study clearly described?	**YES =** if clear inclusion and/or exclusion criteria given.
4. Are the distributions of principal confounders in each group of subjects to be compared clearly described?	**YES** = if age, gender and one or more confounder is described.
5. Are the main findings of the study clearly described?	**YES =** All major findings clearly described so that the reader can check the major analyses and conclusions. Simple outcome data (including denominators, numerators) should be reported for all major findings.<br>**NO =** If only percentages or only p values are reported for main findings.
6. Does the study provide estimates of the random variability in the data for the main outcomes?	For non-normally distributed data the inter-quartile range of results should be reported. In normally distributed data the standard error, standard deviation or confidence intervals should be reported.<br>**YES** = (mean + SD/SE/CI) OR (median + range/IQR).<br>**NO** = mean + range/IQR OR if variability not reported.
7. Have the characteristics of patients lost to follow-up (or non-responders) been described?	**For studies including follow-up:**<br>**YES** = if no patients lost to follow-up; **or** <5% loss to follow-up; **or** tells us something about those lost to follow-up (such as age, gender, that they did not differ from the rest of the cohort etc.). Note, stating the reasons why they were lost to follow-up is not sufficient.

	**NO** = if not explicitly stated how many lost to follow-up **or** does not say anything about the characteristics of those lost to follow-up.<br><br>**For cross-sectional studies**:<br>**YES** = if response rate >80%; **or** tells us something about non-responders (such as age, gender, that they did not differ from the rest of the cohort etc.). Note, stating the reasons why they did not respond is not sufficient.<br>**NO** = if response rate <80% **and** does not explicitly state response rate **and** does not say anything about the characteristics of the non-responders
8. Have actual probability values been reported (e.g. 0.035 rather than <0.05) for the main outcomes except where the probability value is less than 0.001?	Additional explanation not necessary.
9. Were the subjects asked to participate in the study representative of the entire population from which they were recruited?	**YES =** if the source of the population **and** how they were selected is stated; **or** if stated that <u>all</u> of a population were selected; **or** an unselected sample of consecutive patients were selected; **or** a random sample from an entire population were selected.<br>**UTD =** If the study does not state what proportion that sample was from the source population.<br>**NO =** if unable to answer yes to any of the above criteria set for YES or UTD.
10. Were those subjects who were prepared to participate representative of the entire population from which they were recruited?	**YES =** if the study reports how many agreed to participate **and** compares one or more confounder with those agreeing and those not agreeing to participate (ie, that they did not differ from the source population, or mean age, or gender etc.) **OR YES =** if all of a population were selected.<br>**UTD** = if they did not state how many agreed to participate from the source population, or if they did not compare those agreeing with those not agreeing to participate.<br>**NO =** if significant difference was reported in sample compared to population.
11. Was the sample appropriately described with regards to sport-related characteristics?	**YES =** if the type and standard of previous sport participation (professional, college, or recreational) is described **and** either the length of career **or** age at retirement is described
12. Were the main outcome measures used accurate (valid and reliable)?	**YES =** If QOL measures are clearly described, **and** references other article(s) which found outcome measure to be valid and reliable, **or** demonstrates the outcome measure(s) are valid and reliable (note all primary outcomes must be valid and reliable for YES).<br>**NO =** If primary outcomes were not explained in reproducible detail, or validity and reliability not proven/reported.
13. Was there adequate adjustment for confounding in the analyses from which the main findings were drawn?	In non-randomised studies if the effect of the main confounders (gender, age + 1 more) was not investigated **or** no adjustment was made in the final analyses the question should be answered as = **NO**.

	**YES =** If no significant difference in confounders (age, gender + 1 more) between groups stated/shown**. YES =** If adjustments were made for 1 or more confounders in the analysis.
14. Did the study have sufficient power to detect a clinically important effect where the probability value for a difference being due to chance <5%	**YES =** if sample size was calculated and sample size was sufficient to detect a clinically important effect where the probability value for a difference being due to chance <5%.<br>**NO =** if no sample size calculation, **or** calculation found insufficient sample size to detect clinically important effect in primary outcome(s).
